# A New Remote Health-Care System Based on Moving Robot Intended for the Elderly at Home

**DOI:** 10.1155/2018/4949863

**Published:** 2018-02-07

**Authors:** Bing Zhou, Kaige Wu, Pei Lv, Jing Wang, Gang Chen, Bo Ji, Siying Liu

**Affiliations:** ^1^Cooperative Innovation Center of Internet Healthcare, Zhengzhou University, Zhengzhou 450001, China; ^2^School of Information Engineering, Zhengzhou University, Zhengzhou 450001, China

## Abstract

Nowadays, due to the growing need for remote care and the constantly increasing popularity of mobile devices, a large amount of mobile applications for remote care support has been developed. Although mobile phones are very suitable for young people, there are still many problems related to remote health care of the elderly. Due to hearing loss or limited movements, it is difficult for the elderly to contact their families or doctors via real-time video call. In this paper, we introduce a new remote health-care system based on moving robots intended for the elderly at home. Since the proposed system is an online system, the elderly can contact their families and doctors quickly anytime and anywhere. Besides call, our system involves the accurate indoor object detection algorithms and automatic health data collection, which are not included in existing remote care systems. Therefore, the proposed system solves some challenging problems related to the elderly care. The experiment has shown that the proposed care system achieves excellent performance and provides good user experience.

## 1. Introduction

Due to the rapid increase of elderly population, China has become an aging society. At the same time, young people must go to work, which shortens human resource for elderly health care. The population report [1] indicates that the average age in the world increased from 23.5 in 1950 to 28.5 in 2010 and it is expected that it will increase from 29 to 36 between 2013 and 2050 and to 41 in 2100. The proportion of older people (people older than 60 years) increased from 9.2% in 1990 to 11.7% in 2013, and it will reach 21.1% by 2050. Globally, 40% of people older than 60 years live independently, with their spouse only, and it is expected that this number will increase in the future. These “empty-nest” people can easily get into trouble at their homes because they can suffer from sudden health problems.

Currently, there are two main problems related to elderly health care: a real-time communication with family or doctors and a proper mothering. Due to the hearing loss or decreased movement ability, the elderly can find it difficult to conduct a video call because they might not know how to conduct a real-time communication. For instance, it will be very dangerous if an elderly tumbles while he/she is alone at home. Therefore, a continuous and sustainable remote care system for the elderly at home is urgently needed.

In general, there are two kinds of robots used for general care. The first kind are child robots [2], which were made mainly to look after the children. They usually have quick action or naive interaction which might scare or frustrate the elderly. The other kind are professional health-care robots, which are mainly very large and they are commonly used in hospitals. Although these robots can provide satisfactory care, they are very costly and most families cannot afford them. Moreover, common indoor rooms do not have enough space for them.

**Figure 1:**
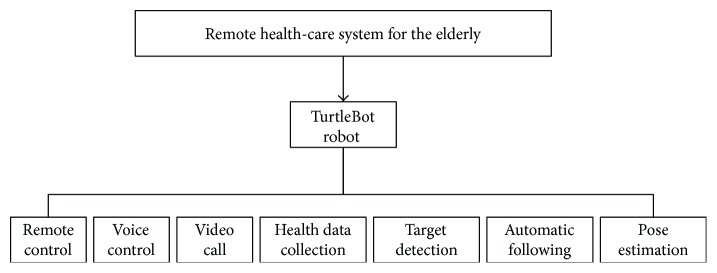
Block structure of the remote health-care system based on moving robot intended for elderly at home.

With the aim of solving the mentioned problems, a remote health-care system based on moving robots intended for the elderly at home is proposed. The main functions of the proposed system are shown in Figure 1. In contrast to the existing care systems, the proposed care system integrates the following practical and fundamental functions:

- The elderly can control robot movement and call their families or community physicians by video/voice.
- The family can remotely control robot movement and conduct video calls with the elderly via mobile phones.
- The system supports accurate object detection and pose estimation. On the one hand, the posture of the elderly can be monitored; namely, the system will automatically alarm in case the elderly tumbles and the elderly can also control the robot through postures.
- The health data can be collected and transmitted to the cloud by sensors connected to the robot. These historical health records can provide very important reference to doctors.

## 2. Related Work

In this section, the review of related remote health-care systems is provided and their drawbacks are discussed.

### 2.1. Remote Health-Care System

Due to the gradual increase of the elderly population, the use of novel technologies in remote health-care systems, as assistant robot systems such as Care-O-Bot [3], Pearl [4], and HOBBIT [5], has increased. These systems are equipped with tablets, sensors, and other devices, and they can provide various services such as speech interaction and video call.

### 2.2. Speech Recognition and Voice Interaction

The automatic speech recognition technology proposed by IEEE [6] effectively reduces errors in speech recognition.

De Wachter et al. [7] presented a speech recognition technology based on template matching; namely, they attempted to overcome voice recognition problems using the straightforward template matching. Maier-Hein et al. [8] put forward a speech recognition technology based on muscle electrical signal. Nevertheless, this method is too difficult to be used by elderly at home. Yu et al. [9] attempted to improve the accuracy of speech recognition using the depth neural network model.

### 2.3. Video Call

Yu et al. [10] proposed a method for transmission of high-quality video signals through Wi-Fi network. They studied three popular mobile video-call applications: FaceTime, Google Plus' Hangouts, and Skype. Lewcio et al. [11] presented a technology for coding and decoding of video signals, which is applicable to all video calls. He proposed a technique for replacement of video codec during real-time video transmissions. Zhang et al. [12] developed a method for adjusting the video call quality in different network environments based on the rate control. They studied the rate control and video quality of Skype video calls and analyzed their network impacts on large-scale networks. Goudarzi et al. [13] put forward a base model wherein the call quality can be predicted. They objectively predicted audio and video calls in wireless applications.

### 2.4. Pose Estimation

There are many works on pose estimation of a single person, which range from simple part detectors and elaborate body models [14, 15] to tree-structured pictorial structure models with strong part detectors [16–18]. In the proposed system, pose estimation relies on integer linear programming of assemble body-part candidates into valid configurations [19].

## 3. Design of Remote Health-Care System

**Figure 2:**
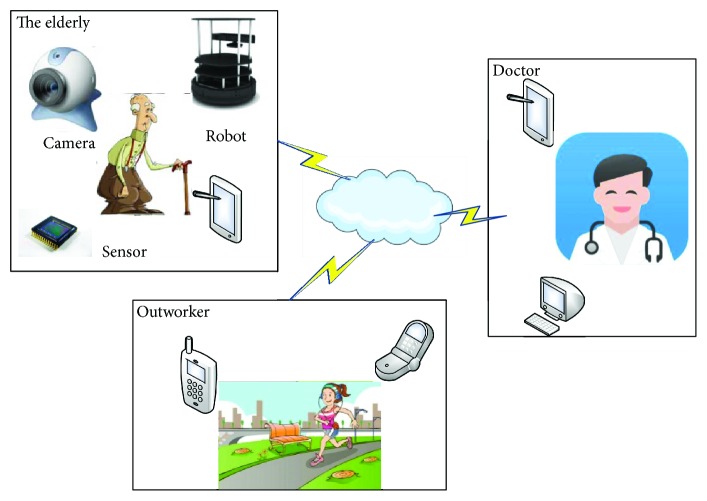
The graphical overview of the proposed system. The health data of the elderly can be collected by sensor connected to the robot at home, and the results can be transmitted to the tablet and stored on the cloud. The health data can be remotely accessed by community doctors. The face and posture of elderly can be detected and analyzed by moving robot. The instructions such as moving forward and back and alarm warning can be conducted by moving robot. The robot motion can be controlled by voice. The video/audio call can be performed between elderly, outworkers, and community doctors.

In this section, the system framework that includes both hardware and software is introduced and design goals are presented. In addition, it is described how the voice, health data, audio/video, and target/pose are detected, processed, and transmitted. The graphical overview of the proposed system is shown in Figure 2. The four main functions of the proposed care system are as follows:

1. The elderly can control robot movement and call families or community physicians by voice.
2. The outworkers can remotely control robot movement and conduct video calls via mobile phones.
3. The proposed system is able to detect the target and estimate its pose, which can determine the elderly status. Moreover, it can automatically alarm when the elderly tumbles. Lastly, the robot can automatically follow the elderly.
4. The health data of old people can be quickly collected and transmitted to the cloud using different sensors that can record the historical health conditions of the elderly, which can help the doctor to judge the condition of the elderly.

## 4. Hardware Architecture

**Figure 3:**
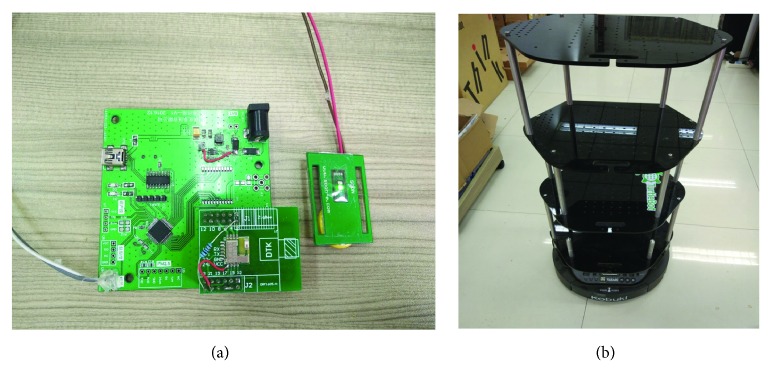
Hardware devices: (a) heart rate sensor and (b) TurtleBot.

The hardware system mainly consists of physical devices, such as heart rate sensor (Figure 3(a)) and TurtleBot (Figure 3(b)).

The heart rate sensor is placed close to the elderly, and its main function is to test the health condition of the elderly continuously. Gao et al. [20] proposed a health data processing algorithm based on partition tuning-based skew handling (PTSH), which improved health data analysis efficiency. The elderly can put their fingers on the sensor, and it will transmit the heart rate of the elderly on the cloud through Wi-Fi. In this way, the health condition of the elderly can be controlled by the outworkers and the doctors.

Hitherto, there have been two generations of TurtleBot. The second generation uses Kobuki (shown in Figure 3(b)) as a control base. In terms of software, the products are developed based on the robot operating system (ROS). The TurtleBot is placed at home, and families can connect to TurtleBot using their mobile phones; thus, they can control robot movement. Additionally, TurtleBot can be controlled by elderly voice.

## 5. Software Design

**Figure 4:**
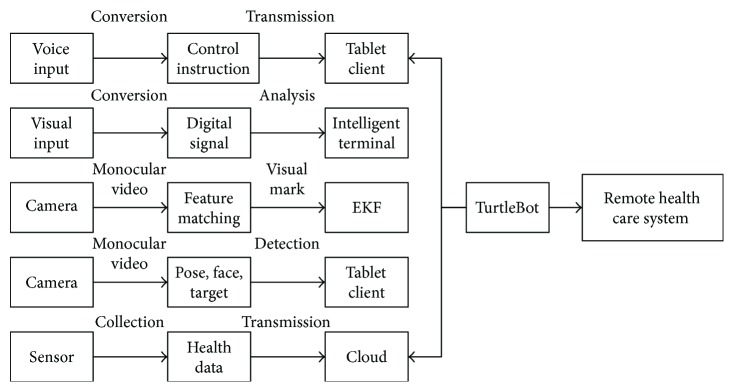
The flowchart of software framework of the proposed remote health-care system.

The software subsystem used in the proposed care system improves quality of traditional elderly-care robots. Namely, it combines video calls, voice control, health data collection, automatic following, target/face detection, and pose estimation. The specific flowchart is shown in Figure 4.

### 5.1. Voice Recognition

The elderly can inevitably face with problems related to the use of the robot. Currently, it is common that robots operate by voice. Therefore, the elderly can control robot motions and conduct video calls by voice.

The spoken speech generally contains nonspeech sounds, such as pause, cough, and environmental noise, which enhances difficulties in the traditional recognition system.

In the field of home care for the elderly, the primary problem relates to the performance improvement of existing speech recognition systems. The confidence model can be an effective solution. The confidence values can be utilized to hypothesize and test the reliability of recognition result and to locate errors in recognition result, which can improve recognition rate and system robustness.

Confidence refers to the probability of correct operation. The confidence value is a measure of mentioned probability, and it indicates the event reliability. In speech recognition, confidence is defined as a function of matching degree between model data and observed data, and it is defined as function *C*(*x*), where *X* is the factor of event space {*x*_1_, *x*_2_,…, *x*_*k*_}. Function *C*(*x*) satisfies the following condition: if the occurrence reliability of *x*_1_ is higher than that occurrence reliability of *x*_2_, then *C*(*x*_1_)≻*C*(*x*_2_), the speech model is set as *W*, and observed speech is *O* = {*o*^1^, *o*^2^,…, *o*^*n*^,…, *o*^*N*^} and the confidence value of *O* relative to *W* is *C*(*O* | *W*). Hence, speech *O* represents the reliability of speech model *W*, which can be used to judge whether the recognition result is correct or not. We can also calculate the confidence value from the perspective of pattern recognition. If we set model *W* as the class 1 and all additional models as the class 2 that is labeled as $\bar{W},$ then we set the identification function *D*(*O*) which satisfies

(1) $$\begin{array}{c} D\left(O\right)\geq 0,\;O\in W,\\ D\left(O\right)\leq 0,\;O\in \bar{W}. \end{array}$$

The identification function is equivalent to the confidence value (*O* | *W*) = *D*(*O*).

According to the confidence level of speech recognition, the confidence model can be divided into preprocessing model, integrated model, and postprocessing model.

Namely, confidence value can be utilized to judge the input signal before recognition. If an input signal does not match with any models, that can be caused by a low signal-to-noise ratio (SNR). In that case, the best solution is to label the confidence value and request for the possible instructions to the elderly. In addition, confidence value can be also utilized to distinguish sex, age, and accent of a person. Consequently, the robot could only receive the instructions from the elderly at home and it would not respond to the instructions from other people. The automatic speech recognition results are hypothesized and tested by confidence value in order to verify the speech. The complete speech verification system should include the following steps.

#### 5.1.1. Increase the User Experience by Engagement

The recognition system can recognize limited number of words. Namely, the speaker might speak words beyond the vocabulary. Moreover, there might be the sounds of breath and cough. The surrounding environment can also generate some sudden noise, and all these sounds are collected by the system. If the system fails to judge the sounds correctly, the output is wrong. Consequently, the system might indicate an incorrect direction and in that case due to the confidence value system effectively requests for the possible instructions which the system missed beyond the vocabulary, which increase the user experience enormously by engagement.

#### 5.1.2. Keywords Determination

The keywords are recognized by the following steps. Firstly, the system recognizes the speech and divides the speech strings into different categories based on grammatical analysis. Secondly, the template matching distance of keywords is calculated, which denotes the keyword detection. Thirdly, the confidence values of selected keywords are estimated and determined, which denote the keyword determination, which can further reduce the probability of true keyword loss and wrong keyword determination. The keywords used in our system are presented in Table 1.

#### 5.1.3. Complete Pronunciation Verification

By establishing the online model and artificial neural network, the confidence values of entire words or characters at different structure levels can be obtained. The confidence values at different levels are accumulated. Then, the confidence value of a complete sentence is obtained, and the complete pronunciation is verified.

**Figure 5:**
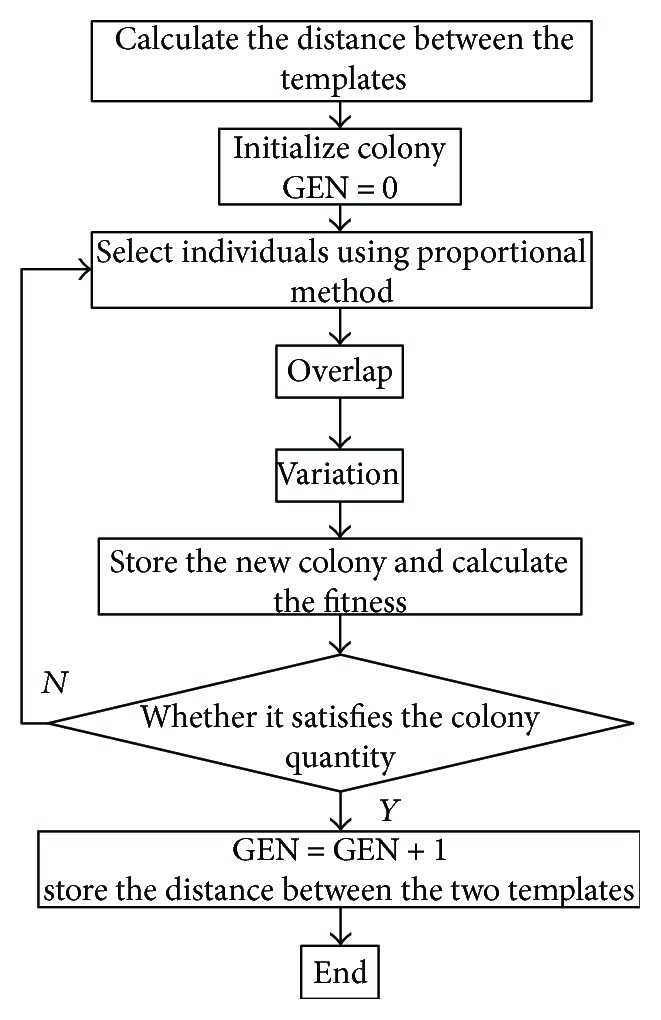
The flowchart of GA_DTW.

By researching voice recognition, we find that there are three popular algorithms: dynamic time warping (DTW), hidden Markov model (HMD), and artificial neural network (ANN). DTW is easy to understand and is suitable for the recognition of isolated words, but it needs a lot of calculation quantity. HMD is complex and needs a lot of training to get reference templates, but it clearly describes voice signal generation processes. ANN has no advantage on voice recognition without combining with other voice recognition algorithms. In the proposed voice system, we mainly recognize keywords, so DTW is feasible. Considering its drawbacks, we study the voice recognition algorithms and propose a new way named Genetic Algorithms Dynamic Time Warping (GA_DTW). Its concrete steps are shown in Figure 5. We compare linear prediction cepstrum coefficient (LPCC) with Mel frequency cepstrum coefficient (MFCC) and select MFCC as a feature parameter because it requires less calculation and more convenient to implement. The aim of DTW is to find a best path to reflect the relationship between reference temple and speech. But GA_DTW abandons the method, and it adopts genetic algorithm to find the best matching path.

Based on above voice interactive technology and system design, after an appropriate training, the movement and video calls of robots can be controlled by a voice, which is very convenient for elderly at home; thus, they are more encouraged to use the robot.

### 5.2. Video Call

In the proposed system, the communication software is installed both on mobile phone and tablet. The mobile phone is held by outworker, and tablet is placed on TurtleBot or held by doctors. The complete process of video call includes registration, launch, maintenance, termination, and cancellation. In the meantime, the remote-control instructions can be also transmitted to tablet; therefore, using this interface, the robots can conduct various motions, such as moving forward or backward and turning left or right.

### 5.3. Pose Estimation

The traditional nursing robots do not have the ability to detect a tumble or to estimate a posture of elderly. From the perspective of telenursing, we tried to design our system such that it can detect the posture of the elderly in real time and make the corresponding responses. For instance, if an elderly tumbles, the robot should automatically alarm and notify outworkers or doctors.

**Figure 6:**
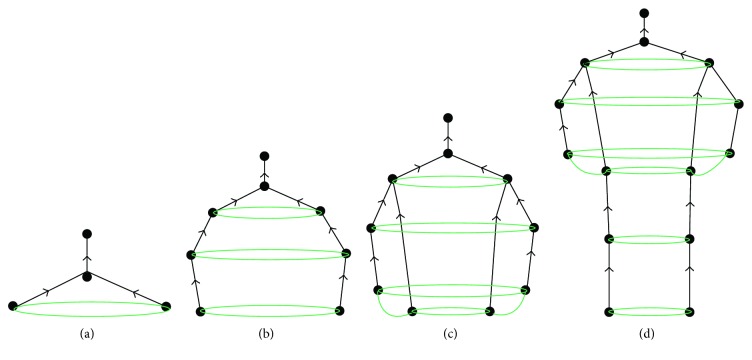
Body joints of posture estimation system.

At present, the postures are mainly estimated based on the graph structure model, which is based on the assumption that there is a constraint relationship between different parts of the body. Each of the body parts faces with a self-occlusion because camera angle influences the accuracy of pose estimation. If there are many people in front of the camera, there will be both self-occlusion and other occlusions, so the fundamental graph structure model cannot meet the requirement for the real-time pose estimation. However, the proposed system firstly detects body-part candidates using a fully connected Convolutional Neural Network (CNN) based on ResNet, and then it employs the integrated linear programming to label and cluster the candidates. The aim of labeling is to mark the candidate's body class, such as the shoulder and head. The aim of clustering is to confirm whether both candidates belong to the same person. At the end, the body joints are divided into three subsets: {head, shoulder}, {elbow, wrist}, and {hip, knee, ankle}. The subset {hip, knee, ankle} is further divided into two parts: {hip} and {knee, ankle}. Firstly, three main subsets are considered, then the head and shoulder (more stable), and lastly the occlusion relationship model is employed. The occlusion diagram is extended based on tree structure. It considers both joint occlusion and abundant occlusion relationship between contextual information and joint. Then, we form a preliminary occlusion relationship structure model (Figure 6(a)). With the aim to ensure proper recognition of the head and shoulder, we add the elbow and wrist into our system (Figure 6(b)). At the end, the hip, knee, and ankle are added as it is shown in Figures 6(c) and 6(d).

#### 5.3.1. The Candidate Choice

**Figure 7:**
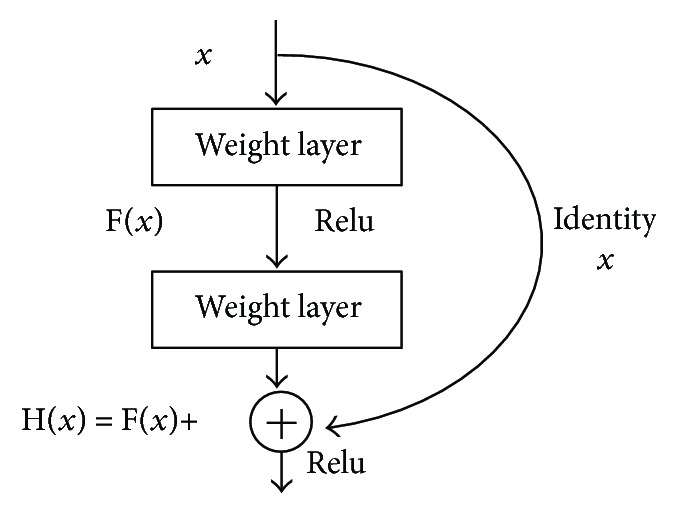
ResNet network structure.

We use two steps to implement the posture estimation. Firstly, we need a stable body detector and a bounding box to label an approximate person location. Then, we analyze the body posture. However, since there is an occlusion among people, detected bounding boxes often exhibit overlaps, which influence the posture estimation. Instead of using people as a detector, a depth fully convolutional human body detection model named ResNet is employed. In contrast to the previous models, such as AlexNet, VGG, and GoogleNet, ResNet can detect a body posture. The body-part detection model has up to 152 layers (Figure 7).

#### 5.3.2. Integer Linear Programming (ILP)

After all body candidates *D* are chosen, we adopt the partitioning and labeling based on the integer linear program. Firstly, we label all candidates, which denotes determination of a body-part class *C* that candidates belong to (e.g., head, shoulder, and knee). Secondly, we determine whether two different candidates belong to one person. Namely, each candidate *d* ∈ *D* and body-part class *c* ∈ *C* have a corresponding unary score, and based on these unary scores, the system associates cost or reward *∂*_*dc*_ ∈ *R* to all feasible solutions for pose estimation. In addition, if there is a relationship between two different candidates, *d* and *d*′, and two body-part classes, *c* and *c*′, then the relationship adopts cost or reward *β*_*dd*′*cc*′_ ∈ *R*. The body parts *d* and *d*′ belong to one person, *d* belongs to class *c* and *d*′ belongs to class *c*′. According to above settings, we can use triple (*x*, *y*, *z*) to indicate it. If *x*_*dc*_ = 1, then the body-part candidate *d* belongs to class *c*; otherwise, it is refrained. Moreover, if *y*_*dd*′_ = 1, then body-part candidates *d* and *d*′ belong to one person. In addition, *z*_*dd*′*cc*′_ = *x*_*dc*_*x*_*d*′*c*′_*y*_*dd*′_ belongs to an auxiliary variable than connects *x* and *y*; thus, *z*_*dd*′*cc*′_ = 1 indicates that candidate *d* belongs to class *c* and that *d* and *d*′ belong to one person. In order to limit each triple to just one body joint, we adopt three linear characteristics.

(1) Uniqueness:
  (2) $$\begin{array}{c} \forall d\in D:\sum_{c\in C}x_{dc}\leq 1. \end{array}$$
(2) Compatibility:
  (3) $$\begin{array}{c} \forall dd^{\prime }\in \left(\begin{array}{l} D\\ 2 \end{array}\right)\text{: }y_{{dd}^{\prime }}\leq \sum_{c\in C}x_{dc},\\ y_{{dd}^{\prime }}\leq \sum_{c\in C}x_{d^{\prime }c}. \end{array}$$
(3) Transitivity:
  (4) $$\begin{array}{c} \forall dd^{\prime }d^{\prime \prime }\in \left(\begin{array}{l} D\\ 3 \end{array}\right)\text{: }y_{{dd}^{\prime }}+y_{d^{\prime }d^{\prime \prime }}-1\leq y_{{dd}^{\prime \prime }}, \end{array}$$
 where *p*_*dd*′*cc*′_ indicates the paired relationship of body structure model and *p*_*dd*′*cc*′_ and *β*_*dd*′*cc*′_ are in the relationship defined by
  (5) $$\begin{array}{c} \beta_{{dd}^{\prime }{cc}^{\prime }}=\log\frac{1-p_{{dd}^{\prime }{cc}^{\prime }}}{p_{{dd}^{\prime }{cc}^{\prime }}}. \end{array}$$

In summary, the posture estimation is based on definition of joint points using 0/1 variable, body-part classes divided into various subsets, and occlusion relationship structure model, which not only optimizes ILP but also solves the occlusion.

#### 5.3.3. Occlusion Relationship Graph Model (ORGM)

In the graph structure model, *G* = (*V*, *ε*) is used to indicate the graph, wherein *N* is the number of nodes and *V* indicates all body parts. After we get the body-joint candidates from an input image, then we use ILP to divide and classify them, and ORGM to limit the relationship between them. Since a lot of time and calculations are needed to consider all candidates, we divide candidates' determination in few steps and use the occlusion relationship structure model. The specific steps, which were previously explained, will be demonstrated on the following example.

**Figure 8:**
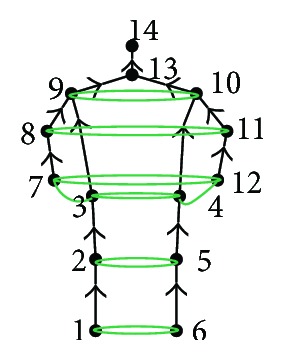
Defined body joints of posture estimation.

Suppose that we have a certain input image. Firstly, we consider the head and shoulder, because as we already mentioned they are stable. Then, we adopt NMS in score map to select the candidates for the head and shoulder, but since we do not know whether they belong to one person or not, we use ILP to label the candidates. In that way, the head (nodes 13 and 14 in Figure 8) and the shoulder (nodes 9 and 10 in Figure 8) are determined. Now, we connect joint 14 and 13, and joint 13 and 9, while joint 10 is used to solve the problem of occlusion. Since, the positions of the head and shoulder are accurate, we begin to consider the elbow (nodes 8 and 11 in Figure 8) and wrist (nodes 7 and 12 in Figure 8). Then, we connect adjacent joints: shoulder (nodes 9 and 10 in Figure 8), elbow (nodes 8 and 11 in Figure 8), and wrist (nodes 7 and 12 in Figure 8). Consequently, the information is transmitted and the occlusion problem is solved.

The rest joints are hip (nodes 3 and 4 in Figure 8), knee (nodes 2 and 5 in Figure 8), and ankle (nodes 1 and 6 in Figure 8). Firstly, we add hip into the system, because hip is an important point that connects the upper part and the lower part of the body; then we add hip into the occlusion relationship structure model and connect the joints. At the end, we choose the candidates for the knee and ankle.

The human motion database of the Weizmann Academy of Sciences is one of the most commonly used for human posture estimation. The current video database containing six types of human actions (walking, jogging, running, boxing, hand waving, and hand clapping) performed several times by 25 subjects in four different scenarios: outdoors, outdoors with scale variation, outdoors with different clothes, and indoors as illustrated below. Because the proposed system aims to estimate the pose of the elderly at home, we select indoors as our video database. Firstly, we input an image into our system to detect body-part features. Then, our algorithm will conduct structure feature learning. Next, the body parts such as the shoulder, ankles, and head will be estimated and their joints will be linked to avoid occlusion. At the end, the person's postures are estimated, the robot can recognize them and people can control robot using posture.

### 5.4. Target/Face Detection and Automatic Following

In order to detect the distance between person and robot, we implemented target detection into our system. The target detection system is based on faster region-based convolutional neural network (Faster R-CNN) algorithm which can detect person quickly. By using this algorithm, we can detect elderly quickly, and human behavior recognition is more accurate.

Furthermore, our system can conduct automatic following based on target detection. TurtleBot utilizes information captured by monocular video to obtain information on target in order to determine the distance between robot and target. In addition, TurtleBot can be driven to move toward the target. At the same time, the improved artificial potential method is adopted for route planning and obstacle avoidance. After the monocular camera captures the target, the host computer analyzes the collected image information and judges whether there are obstacles. If there are obstacles, the corresponding obstacle avoidance strategies are used. Since the target location changes often, the slave computer asks the host computer for location feedback in accordance with the execution. Then, the host computer adjusts the moving trajectory and control instructions timely. At the meantime, we conduct a target detection experience using six behaviors: walking, jogging, running, boxing, hand waving, and hand clapping; the experience results are shown in Table 2.

## 6. Experimental Results

In this section, we first prove that our system can achieve the functional goal, and then we present its application in the real scene.

**Figure 9:**
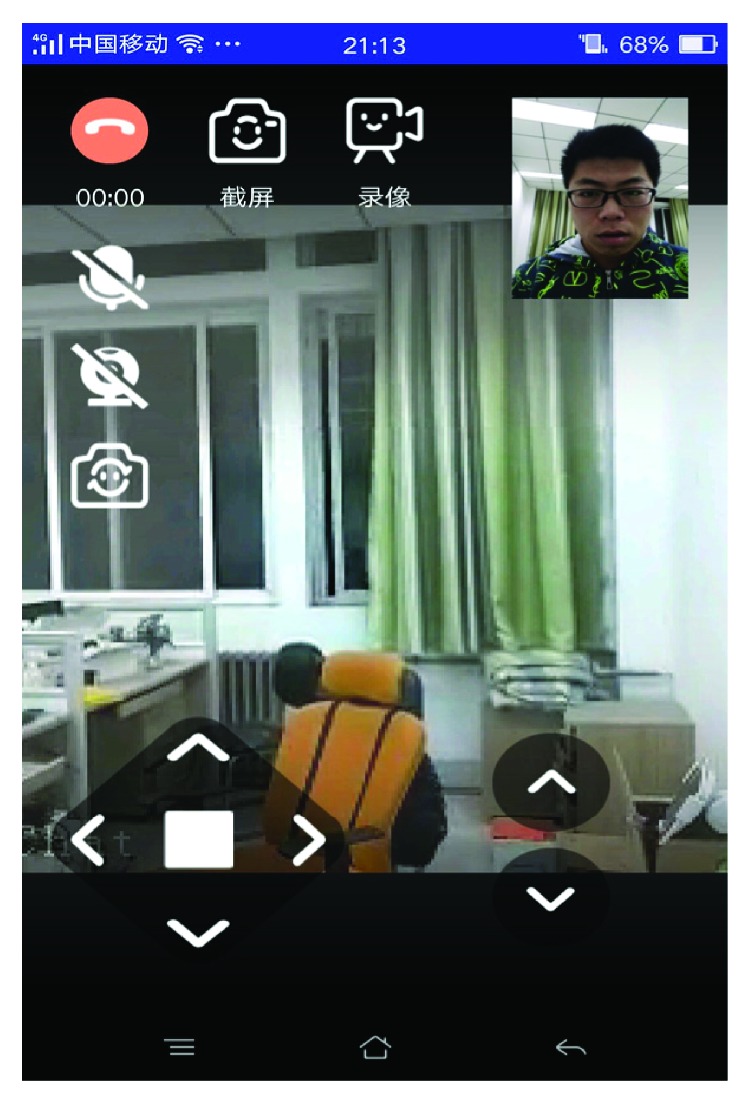
Video call via the remote health-care system.

When an elderly want to conduct video call with others, they can say “Call families” to the robot, then the video call interface will be shown on tablet. In Figure 9, a small window displays the image of the user, and a large screen shows the image transmitted from the tablet on the TurtleBot. The keys placed below the small screen are used to control the robot movement. The outworkers can remotely control the robot and communicate with the elderly via video call.

We conduct an isolated words recognition experience based on DTW and GA_DTW. The words are numbers 0, 1, 2, 3, 4, 5, 6, 7, 8, 9, and 10. We adopt MFCC as a speech feature parameter. The results are shown in Table 3. We can know from the result that DTW's average recognition rate is 89.09% and GA_DTW's average recognition rate is 95.07%. Therefore, GA_DTW has higher recognition rate than DTW on isolated words recognition.

At the meantime, the speech recognition was verified by experiment with four old people. The confidence values of speech units were labeled. The speech units with high confidence were included in self-adaption, and speech units with poor confidence were not included in self-adaption. The self-adaption results (false rates) are shown in Table 4, wherein it can be observed that the introduction of confidence values can effectively improve unsupervised adaptation.

We conduct a continuous word recognition experience. We select key words such as forward, back, left, right, stop, and call. The results are shown in Table 5. We can know from the table that DTW's recognition rate is 83.42%, and GA_DTW's recognition rate is 90.39%. Although the recognition rate is lower than isolated word recognition, we can know that GA_DTW is more efficient than DTW.

In order to measure the accuracy of pose estimation, the correct rates of average positions and average joint points are shown in Table 6, wherein numbers 1, 2, 3, 4, and 5 represent standing forward, standing on one side, stooping, crouching, and lying, respectively.

**Figure 10:**
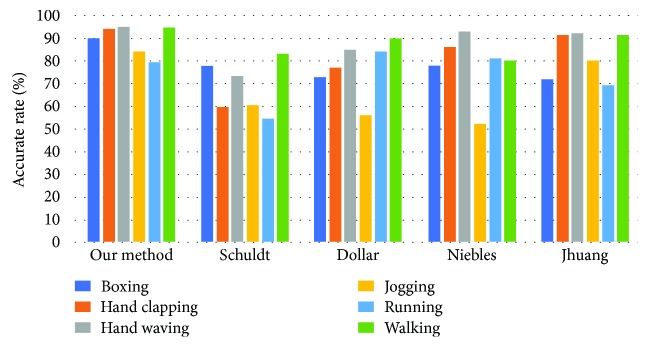
The comparison results between our method and other algorithms.

We conduct a simulation recognition experiment. The database includes six human behaviors: walking, jogging, running, boxing, hand waving, and hand clapping. We put a continuous image of 10 frames. Then, we detect the figures using the proposed algorithms. The contrastive results are shown in Figure 10. By comparing the pose estimation algorithms in other papers, the proposed algorithm has higher recognition accuracy. Therefore, it can play a very good role in practical applications.

We consult a contrast experience between region-based convolutional neural network (R-CNN), fast region-based convolutional neural network (Fast R-CNN), and faster region-based convolutional neural network (Faster R-CNN).

The results are shown in Table 7. From the table, we can know that Faster R-CNN has faster detection speed and higher accuracy than the other two algorithms.

**Figure 11:**
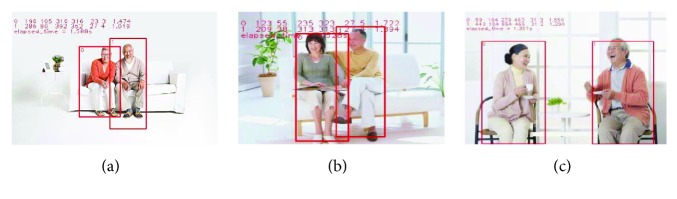
Three detection results for elderly at home. The results show whether there is occlusion or not, the elderly can be detected, and we can get the coordinate information from the detection boxes. Based on the coordinate differences, we can determine person-robot distance indirectly and control the robot by various instructions.

**Figure 12:**
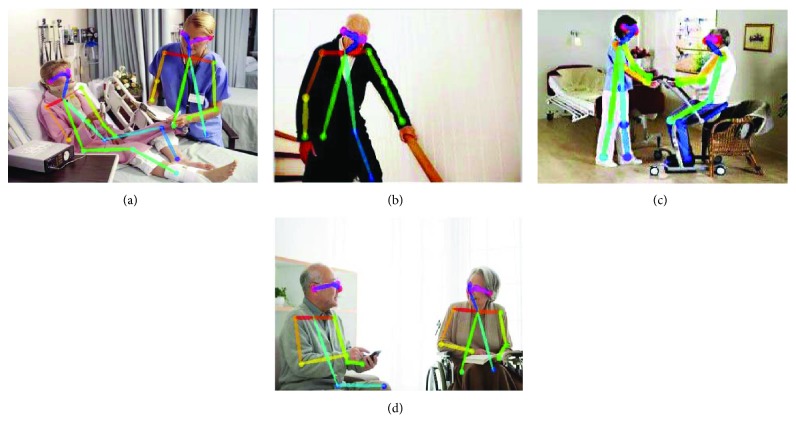
Four posture estimation results in the real-world cases: (a) person who lies, (b) stooped old person, (c) one person who sits and another person who stands up, and (d) persons who sit. According to the posture estimation, when an elderly tumbles, the robot can detect the danger situation automatically and send a warning to the family.

The target detection results are shown in Figure 11, and posture estimation results are shown in Figure 12.

## 7. Conclusion

In this paper, we propose a remote health-care system based on moving robot intended for the elderly at home. The proposed system supports voice control; thus, the elderly can control robot movement by voice and conduct video calls with outworkers and community doctors. Moreover, our system supports remote control, which allows outworkers to control robots remotely via mobile phones. In addition, the health data of the elderly can be collected by heart rate sensor, and health condition of the elderly can be recorded and uploaded to the cloud. Finally, we add posture estimation and face/target detection technology in our system in order to enable it to detect and analyze the posture of the elderly in real time. Based on abovementioned abilities, robot can conduct an automatic following. Moreover, when monitored person come back home, the robot can recognize him and say “Hello.” More importantly, if the elderly tumbles, the robot recognizes the danger and alarms their families or doctors. The experimental results have shown that the proposed system can be used for elderly care. Additionally, it has obvious advantages over the existing remote care systems.

## Figures and Tables

**Table 1:** Keywords.

Number	Instructions
0	Stop
1	Go forward
2	Go back
3	Turn left
4	Turn right
5	Lower the head
6	Raise the head

**Table 2:** The recognition accuracy of target detection.

Human behaviors	Recognition accuracy
Walking	92.5
Jogging	90.5
Running	88.6
Boxing	90.6
Hand waving	91.2
Hand clapping	91.5

**Table 3:** Isolated word recognition results.

Number	DTW (%)	GA_DTW (%)
0	88.56	97.14
1	85.72	91.42
2	91.41	94.30
3	91.43	91.47
4	85.71	94.25
5	91.45	97.14
6	88.55	94.27
7	82.88	91.45
8	88.52	94.33
9	91.48	94.25
10	94.29	97.14

**Table 4:** Self-adaption performance with and without confidence values (false recognition rate).

Speaker	Basic performance (%)	Unsupervised (%)	Supervised (%)	Confidence + unsupervised (%)	Confidence + supervised (%)
1	29.5	19.8	11.1	16.2	11.1
2	45.2	29.5	24.2	27.1	21.4
3	29.5	23.1	19.1	19.1	16.5
4	41.5	28.5	26.3	25.4	24.6

**Table 5:** Continuous word recognition results.

	DTW (%)	GA_DTW (%)
Forward	85.71	92.14
Back	82.14	88.52
Stop	83.74	89.98
Left	81.14	92.18
Right	85.85	89.88
Lower	84.12	93.35
Raise	85.14	94.12
Open	84.12	87.98
Close	85.12	89.24
Video	84.46	89.88
Charge	79.88	86.82
Walking	85.55	91.23
Out	84.45	92.12
Sing	78.88	86.32
Weather	79.68	92.11
Up	83.31	90.51
Down	83.35	90.53
Voice	83.30	90.49
Pose	83.36	90.55
Call	84.10	90.15
Song	84.40	90.18

**Table 6:** The comparison of recognition accuracy.

Actual posture	Average position (%)	Average joint point (%)
1	77.2	65.2
2	76.8	68.4
3	71.1	61.2
4	70.1	60.5
5	78.8	63.9

**Table 7:** Speed/accuracy contrast experience.

	R-CNN	Fast R-CNN	Faster R-CNN
Test time per image	50 s	2 s	0.2 s
Speedup	1x	25x	250x
mAP (VOC2007)	66.0	66.9	73.2
